# Acquisition of HIV by African-Born Residents of Victoria, Australia: Insights from Molecular Epidemiology

**DOI:** 10.1371/journal.pone.0084008

**Published:** 2013-12-31

**Authors:** Chris Lemoh, Claire E. Ryan, Zamberi Sekawi, Anna C. Hearps, Eman Aleksic, Doris Chibo, Jeffrey Grierson, Samia Baho, Alan Street, Margaret Hellard, Beverley-Ann Biggs, Suzanne M. Crowe

**Affiliations:** 1 Centre for Population Health, The Burnet Institute, Melbourne, Victoria, Australia; 2 Department of Medicine, The University of Melbourne, Melbourne, Victoria, Australia; 3 Victorian Infectious Disease Service, Royal Melbourne Hospital, Melbourne, Victoria, Australia; 4 Centre for Biomedical Research, The Burnet Institute, Melbourne, Victoria, Australia; 5 Faculty of Medicine and Health Sciences, University Putra Malaysia, Serdang, Selangor, Malaysia; 6 HIV State Reference Laboratory, Victorian Infectious Disease Reference Laboratory, Melbourne, Victoria, Australia; 7 Australian Research Centre in Sex, Health and Society, La Trobe University, Melbourne, Victoria, Australia; 8 Department of Infectious Diseases, Alfred Hospital, Melbourne, Victoria, Australia; University of Cincinnati College of Medicine, United States of America

## Abstract

African-born Australians are a recognised “priority population” in Australia's Sixth National HIV/AIDS Strategy. We compared exposure location and route for African-born people living with HIV (PLHIV) in Victoria, Australia, with HIV-1 *pol* subtype from drug resistance assays and geographical origin suggested by phylogenetic analysis of *env* gene. Twenty adult HIV positive African-born Victorian residents were recruited via treating doctors. HIV exposure details were obtained from interviews and case notes. Viral RNA was extracted from participant stored plasma or whole blood. The *env* V3 region was sequenced and compared to globally representative reference HIV-1 sequences in the Los Alamos National Library HIV Database. Twelve participants reported exposure via heterosexual sex and two via iatrogenic blood exposures; four were men having sex with men (MSM); two were exposed via unknown routes. Eight participants reported exposure in their countries of birth, seven in Australia, three in other countries and two in unknown locations. Genotype results (*pol*) were available for ten participants. HIV *env* amplification was successful in eighteen cases. HIV-1 subtype was identified in all participants: eight both *pol* and *env*; ten *env* alone and two *pol* alone. Twelve were subtype C, four subtype B, three subtype A and one subtype CRF02_AG. Reported exposure location was consistent with the phylogenetic clustering of *env* sequences. African Australians are members of multiple transnational social and sexual networks influencing their exposure to HIV. Phylogenetic analysis may complement traditional surveillance to discern patterns of HIV exposure, providing focus for HIV prevention programs in mobile populations.

## Introduction

African-born Australians have been recognised as a “priority population” in Australia's Sixth National HIV/AIDS Strategy, published in 2010 [1]; they are diagnosed late with HIV compared to Australian-born people living with HIV (PLHIV) and are over-represented among new diagnoses of HIV [2]–[4].

National surveillance data indicates that heterosexual sex is the most common exposure route reported among African-born Australians diagnosed with HIV, as it is in populations on the African continent [3], [5], although the proportion of African-born people diagnosed with AIDS in Australia includes a higher proportion of MSM than is reported amongst African migrants in European AIDS surveillance figures [6], [7] and there is growing recognition of the importance of sex between men as an epidemiological factor in low-income countries, including Sub-Saharan Africa [8], [9]. European studies suggest most African-born PLHIV are infected in their countries of origin, prior to migration [10] however Australia is more geographically distant from Africa and has a more regulated migration process that results in the *de facto* exclusion of most prospective immigrants with HIV [11]. All adults and some children applying for permanent residency are screened for HIV, as are some temporary entrants (those working in health care and students from Sub-Saharan Africa intending to stay for at least one year); those diagnosed with HIV are denied a visa except the minority who are granted a waiver of the health requirement [11], [12]. The experience of migration-related HIV screening has led some African Australians to view HIV as a problem left behind when migrating to Australia: a view that is at odds with the surveillance data described above [13]–[15]. When planning HIV prevention and testing strategies it is therefore important to understand when African-born Australians are exposed to HIV with respect to their initial migration, and whether exposure occurs within Australia or abroad.

Some information about HIV exposure in African Australian communities can be obtained through routine passive surveillance procedures, but passive surveillance data is based on information obtained during routine clinical consultation—the accuracy and reliability of which information may be compromised by numerous patient, clinician and structural factors influencing the clinical interaction, during initial and subsequent visits [16]–[18]. More detailed information about individual and population level factors influencing exposure requires active data collection. Interviews, surveys and field observation provide valuable epidemiological data, but molecular techniques such as phylogenetic analysis may provide complementary information to clarify the location and route of HIV acquisition and its timing with respect to migration.

HIV-1 subtype C accounts for more than 50% of all infections globally [19], and dominates HIV epidemics in Sub-Saharan Africa, India, Papua New Guinea, and parts of South America. Two circulating recombinant forms (CRFs), CRF01_AE and CRF02_AG, are each responsible for 5% of HIV cases. In Sub-Saharan Africa the distribution of HIV subtypes is diverse: subtype A is found in western, eastern and central Africa; subtype C in western and southern Africa; subtype G in the western and central regions; and subtype D in the central and eastern regions. CRF02_AG is concentrated in the western region. Other subtypes such as F, H, J and K are mainly found in central Africa [20], [21]. Subtype B is the subtype most commonly reported from Western Europe, North America and Australia, regardless of reported risk factor, although a recent paper describing non-B subtypes in Australia linked both CRF01_AE and subtype C with MSM transmission and reported subtype C to be the most common subtype among females [22].

The ability to describe the genetic similarities and differences between HIV strains has contributed to our understanding of the epidemiology of HIV, when phylogenetic data have been compared with epidemiological data obtained from history or observation of HIV exposure and transmission events. Sources of demographic and epidemiological data have usually included questionnaires completed by treating physicians [23], [24], surveillance notification data [22], [25]–[27], routine clinical case notes [10], [28] or cohort study data [29], [30]. Less commonly, researchers have conducted direct interviews or administered questionnaires to PLHIV to obtain information about travel and exposure [23], [31], [32]. Full genome sequencing has provided the most detailed phylogenetic discrimination, enabling identification of recombinant strains and discriminating between closely related strains [33]; this has been most valuable in outbreak investigations and forensic settings, where it has been necessary to discriminate between closely-related strains isolated from individuals from whom detailed information was obtained about travel, social contacts, sexual and other risk behaviours [32], [34]. However, many phylogenetic studies have used genetic sequencing of *pol* gene conveniently obtained from antiretroviral resistance genotyping undertaken in routine clinical practice to detect mutations in reverse transcriptase (RT) or protease (PR); sequences from recently-infected or drug-naive patients have been preferred, to avoid genetic variation arising from drug selection pressure [24], [27]–[30], [35], [36]. Other researchers have used *gag* or *env* sequences, for which genetic variation is less subject to drug-related selection pressure in PLHIV already on treatment [10], [25], [26]. Limited sequencing of the highly variable region of *env* has provided useful epidemiological information in a range of social and geographical settings [10], [23], [25], [26], [28], [32], [37]–[39].

This study aimed to describe the location and route of HIV infection for African-born residents of Victoria and to compare the self-reported history of HIV exposure with the likely geographical origin of the HIV suggested by phylogenetic analysis. We hope that the results of this study will help inform the development and implementation of strategies to detect prevalent HIV infections amongst African immigrants exposed prior to migration and to prevent incident infections amongst those not already exposed, either through exposure within Australia or during subsequent travel abroad.

## Materials and Methods

### Participant recruitment

A case series of African-born adults living with HIV in Victoria was conducted, in which history of reported exposure to HIV was obtained and examined in the context of phylogenetic analysis of HIV strains isolated from participants. Participants were recruited via treating doctors who informed eligible patients about the study and sought permission to provide contact details to the research team. A member of the research team contacted potential participants to describe the study. Written informed consent was obtained from those agreeing to participate.

### Ethics statement

The study was designed and conducted in consultation with several African community-based organisations. The human research ethics committees of Melbourne Health and Alfred Health Ethics approved the study protocol. Consent was obtained separately and specifically for the research team to access stored blood samples for the purpose of genetic sequencing of HIV and/or for the collection for this purpose of fresh blood via venepuncture, if suitable stored blood samples were unavailable. Participants were reimbursed AUD $25.00. All information was de-identified prior to analysis.

### Participant interviews and review of case notes

Self-reported location of HIV exposure was obtained both directly from interviews, and indirectly from recorded case notes on exposure histories elicited at HIV diagnosis and during subsequent consultations. If country of exposure was not named either in interview or case notes, the location of exposure was recorded as “unknown”. The route of HIV exposure was classified as: male-to-male sex (with or without a history of injecting drug use) (MSM+/−IDU); heterosexual sex; injecting drug use (IDU), and other/unknown. HIV risk factors of source partners were recorded for those participants with a sexual exposure to HIV. Details of circumstances of exposure were obtained from interviews and from case notes. Although year of migration was not specifically asked (due to prevailing political tensions regarding migration of PLHIV) most participants volunteered this information [40], [41]. If participants had undergone a previous HIV genotypic drug resistance assay through one of the two certified Victorian reference laboratories, the genetic information for the *pol* gene was obtained from the relevant databases. In addition, for participants that consented to either access to stored plasma or providing a fresh blood samples *env* subtyping was performed.

Details of HIV-related symptoms and diagnoses were obtained through interview as well as case notes. Results of investigations were obtained from case notes. Late diagnosis was defined as either a CD4 count <350 cells/µL or an AIDS-defining illness at HIV diagnosis; this category included cases of advanced HIV disease at diagnosis, who had a CD4 count <200 cells/µL or an AIDS-defining condition at HIV diagnosis [42]. AIDS-defining conditions were those listed in the surveillance definition of AIDS published by the Centers for Diseases Control and Prevention, other than a CD4 count <200 cells/µL in the absence of opportunistic infection [43].

### Laboratory procedures

Stored participant plasma samples were obtained from the Victoria Infectious Diseases Reference Laboratories (VIDRL) and the Burnet Institute (Victorian HIV Blood and Tissue Storage Bank). Fresh whole blood samples were collected in 10 mL EDTA tubes during venepuncture for routine clinical blood tests.

Viral RNA was extracted from plasma using the QIAamp Viral RNA Mini Kit (Qiagen, Germany) and from whole blood using the High Pure Viral Nucleic Acid kit (Roche, United Kingdom) as per manufacturers' instructions. For all extracts, a nested reverse transcription PCR (RT-PCR) was used to amplify the V3 region of the HIV *env* (corresponding to nucleotides 6959–7374 on HXB2 reference genome, accession number K03455), following the methodology previously used by our team for a phylogenetic study of HIV amongst Vietnamese-Australian people injecting drugs [44], [45]. Briefly, the reverse transcriptase and first 35 amplification cycles was performed using the OneStep RT-PCR kit (Qiagen, Germany) and 0.6 µM of outer primers, EnvF1 and EnvR1, which corresponded to nucleotides 6847–6871 and 7365–7389, respectively. 10 µl of template RNA was added to a final reaction volume of 25 µl before cycling commenced. As per manufacturers instructions, cycling parameters included an initial step of 50°C for 30 minutes to allow for reverse transcription, prior to the DNA polymerase activation step of 95°C for 15 minutes. Thirty-five cycles of 94°C for 30 secs, 40°C for 30 secs and 72°C for 2 minutes and a 10 minute final extension at 72°C completed the amplification.

Nested PCR was performed with 0.5 µM inner primers EnvF2 and EnvR2, corresponding to nucleotides 6959–6983 and 7350–7374 respectively. Platinum Pfx polymerase (Invitrogen) was used according to manufacturers instructions with 2.5 µl of the first round PCR product as template in a final volume of 50 µl. Cycling parameters were as above with the omission of the reverse transcription step. The length of the final product was 415bp. PCR product purification, sequencing and sequence assembly was performed as previously described [25].

### Sequence analysis

To determine subtype, consensus sequences were aligned in MEGA version 5 software suite with reference strains of each HIV-1 group M isolate and a phylogenetic tree constructed to determine subtype (data not shown) [46]. For further analysis, the consensus sequence for each isolate was subject to the Basic Local Alignment Search Tool (BLAST) program within the National Centre for BioInformatics (NCBI) software suite to determine the most closely related published isolates. The three most similar sequences were downloaded. Once final sequences and subtypes were determined, all downloaded sequences, plus a selected reference sequence of each subtype were aligned with the study sequences using CLUSTAL W in MEGA version 5. This alignment was exported and used to infer a neighbour-joining phylogenetic tree on the basis of pairwise genetic distances calculated using the two-parameter algorithm described by Kimura and Bull [47]–[49]. Statistical support for the tree topology was assessed by bootstrap analysis with 1000 replicates [45]. A bootstrap value of 70% or greater was used to define a phylogeny cluster. Sequences were submitted to the GenBank database under the accession numbers GU211906 to GU211923.

### Statistical analysis

Standard descriptive statistical methods were used to describe the demographic and epidemiological characteristics of the participants. Groups were compared using Student's t test or the Wilcoxon rank-sum test for continuous variables and the chi-square test or Fisher's exact test for categorical variables, as appropriate. A p value less than 0.05 was considered statistically significant. The demographic and clinical characteristics of the case series participants, along with some details of HIV exposure, are presented in another manuscript by the authors [50]. Analysis was carried out using the statistical analysis software package Stata 10.

## Results

The Victorian HIV surveillance database (Centre for Population Health, Burnet Institute) indicated that 145 African-born individuals had been notified to the Victorian Department of Human Services (DHS) with newly diagnosed HIV infection by 2005 (Guy R, personal communication: Notifications of HIV diagnosis in Victoria: Country of birth in Africa; extract from Victorian HIV Registry. 2005 February 14). Surveillance records included the name and location of referring doctors and initial specialty clinics to which cases were referred, but no details of subsequent consultations or emigration from Victoria. Preliminary enquiries of the HIV specialty clinics, about newly diagnosed patients referred to their services, suggested that approximately 80 African-born patients with HIV were attending these clinics in 2005. Recruitment was undertaken during 2006 and 2007. Treating doctors approached 36 individuals in the course of their routine clinic visits, of whom 31 agreed to be contacted by the research team. Twenty-two individuals consented to take part in the project. Two withdrew from the study, leaving a small sample of 20 participants. Participants were interviewed between 9 May 2006 and 23 July 2007. Case notes were reviewed between 7 September 2006 and 4 March 2009. The time between HIV diagnosis and interview ranged from six months to 16 years, with a median of 63.5 months.

Fourteen participants were male and six were female. Nine were born in Ethiopia; two each born in Mauritius, Zimbabwe, and Kenya; one each born in Eritrea, Zambia, Ghana, Tanzania and South Africa. Twelve individuals were exposed via heterosexual sex, four via male-to-male sex and two via health-care related blood exposure. None were exposed via IDU. For two participants the route of exposure was unknown (Table 1). The median age at HIV diagnosis was 39 years (range 25 to 51). Median CD4 count at HIV diagnosis was 145 cells/µL, with an interquartile range (IQR) of 60 to 320. Median viral load at diagnosis was 4.76 log_10_ copies/mL (IQR 4.29 to 5.00). One participant was diagnosed during seroconversion. Fourteen were diagnosed late, including ten diagnosed with advanced HIV disease. Ten were asymptomatic at diagnosis; one experienced seroconversion illness; nine were symptomatic, of which five had AIDS-defining illness (Table 2). Eighteen were diagnosed in Australia and two in New Zealand (both before migration to Australia). Five were diagnosed during migration-related HIV screening (two in New Zealand and three in Australia). Eleven had previously tested negative for HIV (five during migration-related screening). Five had most recently tested negative in Australia and six abroad. Seven (50%) of the 14 diagnosed late had previously tested negative, including four (29%) who tested negative in Australia. Nine reported no previous HIV test before diagnosis (Table 1). Fifteen were on antiretroviral therapy at the time of interview. Eighteen (90%) reported their current state of health as “excellent” or “good.” Details of clinical state and treatment have been published elsewhere [50].

**Table 1:** African-born Victorian residents with HIV: participant characteristics, HIV testing history, exposure and HIV-1 subtype.

	Demographic characteristics				HIV testing history		HIV exposure			HIV subtype[1]		Accession No.	Comments on exposure
Case No.	Gender	Age at diagnosis (yrs)	Country of birth	Year of arrival	Year of HIV test		Exposure route	Self-reported exposure location					
					Last negative	First positive		Interview	Case notes	*pol*	*env*		
CS01	M	51	South Africa	Unknown	1987	2001	MSM+/−IDU	Australia/Europe	Unknown	—	B	GU211906	Negative HIV test in Australia 14 years before diagnosis. Multiple male sexual contacts for many years in Australia, Europe.
CS02	M	40	Eritrea	1995	2003	2005	Heterosexual	Kenya/Australia	Unknown	—	C	GU211907	Negative HIV test in Australia 2 years before diagnosis. No details of source partner. Travel history not available.
CS05	M	36	Ethiopia	1994	1994	2002	Heterosexual	Kenya	Unknown	C	C	GU211908	Last ngeative test in Kenya 8 years before diagnosis. Case notes: heterosexual contact with woman from high-prevalence country. Interview: no sexual source mentioned, only low-risk health care-related blood exposures.
CS06	F	27	Kenya	1997	1997	1998	Other	Kenya	Kenya	—	A	GU211909	Last negative test in Kenya 1 year before diagnosis. Multiple health-care related blood exposures in Kenya. Male partner in Kenya HIV negative.
CS07	M	40	Mauritius	Unknown	2004	2004	MSM+/−IDU	Australia	Australia	B	B	GU211910	Documented seroconversion after unprotected male-to-male sex in Australia.
CS08	M	38	Ethiopia	1996	1996	2002	MSM+/−IDU	Australia	Unknown	B	B	GU211911	Negative HIV test in UK 6 years prior to diagnosis. Male-to-male sex in Australia.
CS09	F	32	Zimbabwe	1999	1998	2002	Other	Zimbabwe	Unknown	—	C	GU211912	Two health-care related blood exposures in Zimbabwe after last negative test in Zimbabwe 3 years before diagnosis.
CS10	F	43	Zambia	1989	No prior test	2000	Unknown	Australia	Unknown	—	C	GU211913	One male partner (later found to be HIV+) since 20 years prior to HIV diagnosis. No blood-borne exposure. No previous HIV test.
CS11	M	48	Ethiopia	Unknown	No prior test	2005	Unknown	Ethiopia	Unknown	—	C	GU211914	No exposure history available
CS12	M	34	Kenya	Unknown	No prior test	2006	Heterosexual	Kenya	Kenya	A1	A	GU211915	Heterosexual contact in Kenya with female partner from high-prevalence country.
CS13	F	25	Ethiopia	1998	No prior test	2001	Heterosexual	Unknown	Uganda/Ethiopia	C	—	Not sequenced	Heterosexual contact in Uganda/Ethiopia.
CS14	M	47	Ethiopia	1996	1995	2006	Heterosexual	Australia	Unknown	—	C	GU211916	Negative HIV test in Australia 11 years prior to diagnosis. Unknown source partner.
CS15	M	42	Tanzania	Unknown	No prior test	1995	Heterosexual	Europe	Unknown	—	A	GU211917	Multiple heterosexual contacts in Europe and Tanzania.
CS16	F	27	Ethiopia	Unknown	1990	1997	Heterosexual	Unknown	Ethiopia	C	—	Not sequenced	Possible heterosexual contact or needle exposure in Ethiopia after negative test in Sudan 7 years before diagnosis
CS17	M	41	Ethiopia	1993	1993	2002	Heterosexual	Australia	Unknown	—	C	GU211918	Source partner known HIV+. Negative HIV test in Sudan 11 years prior to diagnosis.
CS18	M	30	Ethiopia	Unknown	No prior test	1998	Heterosexual	Ethiopia	Ethiopia	C	C	GU211919	Heterosexual exposure in Ethiopia.
CS19	M	26	Ethiopia	1990	No prior test	1990	Heterosexual	Ethiopia	Ethiopia	C	C	GU211920	Possible heterosexual or low-risk blood exposure in Ethiopia.
CS20	F	31	Ghana	2005	No prior test	2005	Heterosexual	Ghana	Ghana	AG	A	GU211921	Male sexual partners in Ghana. No other exposure history available.
CS21	M	47	Zimbabwe	Unknown	2000	2002	Heterosexual	PNG	PNG	—	C	GU211922	Heterosexual sex with PNG-born female partner in PNG after last negative test in Australia 2 years before diagnosis.
CS22	M	40	Mauritius	Unknown	No prior test	1992	MSM+/−IDU	Australia	Unknown	B	B	GU211923	Multiple male-to-male sexual exposures in Australia.

[1] *pol* subtype from antiretroviral susceptibility genotype assay; *env* subtype from sequencing and phylogenetic analysis of V3 (see Methods).

**Table 2:** African-born Victorian residents with HIV: stage at diagnosis.

Stage at diagnosis	CD4 count (cells/µL)	Symptoms and (CD4 count) at HIV diagnosis				
		Seroconversion	Asymptomatic	Non-AIDS*	AIDS†	Details of symptoms
	Unknown		CS22‡			
				CS19‡		Genital herpes
	>350		CS21 (996)			—
			CS06 (855)			—
				CS16 (678)		Fever
		CS07 (647)				Seroconversion illness
**Late HIV diagnosis** §	350–200		CS09 (320)			—
			CS15 (250)			—
			CS10 (247)			Appeared unwell to mother
			CS20 (233)			—
**Advanced HIV disease** **	<200		CS17 (150)			—
					CS02 (14)	Non-Hodgkin lymphoma
			CS18 (120)			—
					CS05 (120)	Cerebral toxoplasmosis
			CS11 (118)			—
				CS13 (60)		Shingles
					CS01 (40)	PCP††
					CS08 (30)	Cerebral toxoplasmosis
					CS14 (3)	PCP, CMV,‡‡ oesophageal candidiasis
				CS12 (2)		Pneumonia

Non-AIDS defining condition.

Condition fitting definition of acquired immune deficiency syndrome used in national HIV/AIDS surveillance (Kaldor J & McDonald A. JAIDS. 2003;32 Suppl 1).

CD4 count at HIV diagnosis not recorded.

Late HIV diagnosis: CD4 count <350 cells/µL at HIV diagnosis, including “advanced HIV disease”.

Advanced HIV disease: CD4 count <200 cell/µL at diagnosis or AIDS at HIV diagnosis.

*Pneumocystis jiroveci* pneumonia.

Cytomegalovirus retinitis or disease other than liver, spleen or nodes.

No blood sample was obtainable from CS13. CS16 did not consent to the use of stored or fresh blood for HIV sequencing, but did consent to the use of information from case notes and investigations. Both of these patients had prior evidence of clinical ARV resistance as determined by an HIV drug resistance genotyping assay, from which *pol* sequence results (subtype C) were obtained, but no *env* sequence data were available for phylogenetic analysis.

Interview data named location of exposure in 18 cases (in two of these case, two regions were named), with two cases reporting “unknown” location. Case notes recorded named exposure location in nine cases (in one case, two regions were named), with eleven cases recording “unknown” location. Reported location of exposure from interview and case notes was consistent in seven cases. In the remaining 13 cases, exposure location was named in either interviews or case notes alone. In no case did the location of exposure reported from the interview contradict the case notes. The reported location of exposure were: Australia in six cases; country of birth in eight cases; Australia or country of birth in one case; a third location (other than country of birth or Australia) in three cases; and either Australia or a third location in two cases. Overall, 11 (55%) reported exposure in Africa, eight (40%) in Australia and three (15%) in another region (Europe or PNG) (Table 1).

Ten participants had undergone HIV genotyping for drug resistance testing; genotyping laboratories provided *pol* sequences for all these participants. Fifteen stored plasma samples were accessed and three whole blood samples were collected from participants for *env* sequence analysis. Extraction and amplification from the samples were attempted and amplification and sequencing was successful for all samples.

Subtype was obtained solely from *env* in ten cases and from *pol* alone in two cases. Of the ten *pol* sequences, five were subtype C, three subtype B, one subtype A and one subtype AG. Of the 18 *env* sequences, ten were subtype C; four subtype B and four subtype A. For eight participants, both *pol* and *env* genetic information were available. HIV-1 subtype was identical in seven of these cases; the exception was CS20, where CRF02_AG was reported in the *pol* region and subtype A in the *env* region. This participant was likely to be infected with CRF02_AG, in which the *env* region is almost entirely comprised of subtype A sequence. Taking both *pol* and *env* sequences together, 12 (60%) participants had HIV-1 subtype C, four (20%) subtype B, three (15%) subtype A and one (5%) CRF02_AG (Table 1).

Three participants with subtype A were exposed via heterosexual sex. One (CS12) reported exposure in his country of birth; the second (CS15) had multiple female sexual contacts in Europe and his country of birth. The third participant (CS06) had multiple health-care associated blood exposures in her country of birth. The participant with CRF02_AG (CS20) had multiple heterosexual contacts in her country of birth.

Nine of the 12 participants with subtype C were exposed via heterosexual sex, one (CS09) via health-care associated blood exposure and two (CS11 and CS10) via unknown routes. Three participants reported heterosexual exposure in Australia: one (CS02) had a negative HIV test in Australia two years prior to diagnosis but did not report a specific source partner (he also had heterosexual contact in Kenya, but attributed his HIV infection to exposure in Australia); another (CS14) reported heterosexual contact in Australia and had last tested negative abroad 11 years prior to diagnosis; the third (CS17) had an HIV-positive source partner and had last tested negative to HIV abroad eleven years prior to diagnosis). Five participants (CS05, CS13, CS16, CS18, CS19) reported heterosexual exposure in Africa (CS16 reported exposure in Ethiopia, possibly via heterosexual contact or health care-associated blood exposure; she did not consent to the use of stored or fresh blood samples for genetic sequencing). One participant (CS21) reported heterosexual contact in Papua New Guinea with a local-born female partner. One participant (CS09) reported health care-associated blood exposures in her country of birth and had never been sexually active. One male participant (CS11) reported exposure in Ethiopia via an unknown route. One female participant (CS10) reported exposure in Australia via an unknown route; however she also reported that her only sexual partner for the twenty years preceding her diagnosis, who had migrated with her from Africa to Australia several years previously, had been diagnosed with HIV after migration; she denied any exposure to blood products and had not previously been tested for HIV; the likelihood appears strong that she had in fact acquired HIV from her long-term male partner, before or after migration.

All four participants with subtype B viruses were exposed via male-to-male sex. One (CS07) was certainly exposed in Australia, having a documented seroconversion after a high-risk sexual contact. Two were likely exposed in Australia, reporting multiple male sexual contacts in Australia; one (CS08) had a negative HIV test abroad six years prior to his diagnosis and the other (CS22) had not previously been tested for HIV. The remaining man (CS01) had multiple male sexual contacts in Europe and Australia and had not been tested for HIV prior to his diagnosis.

**Figure 1:**
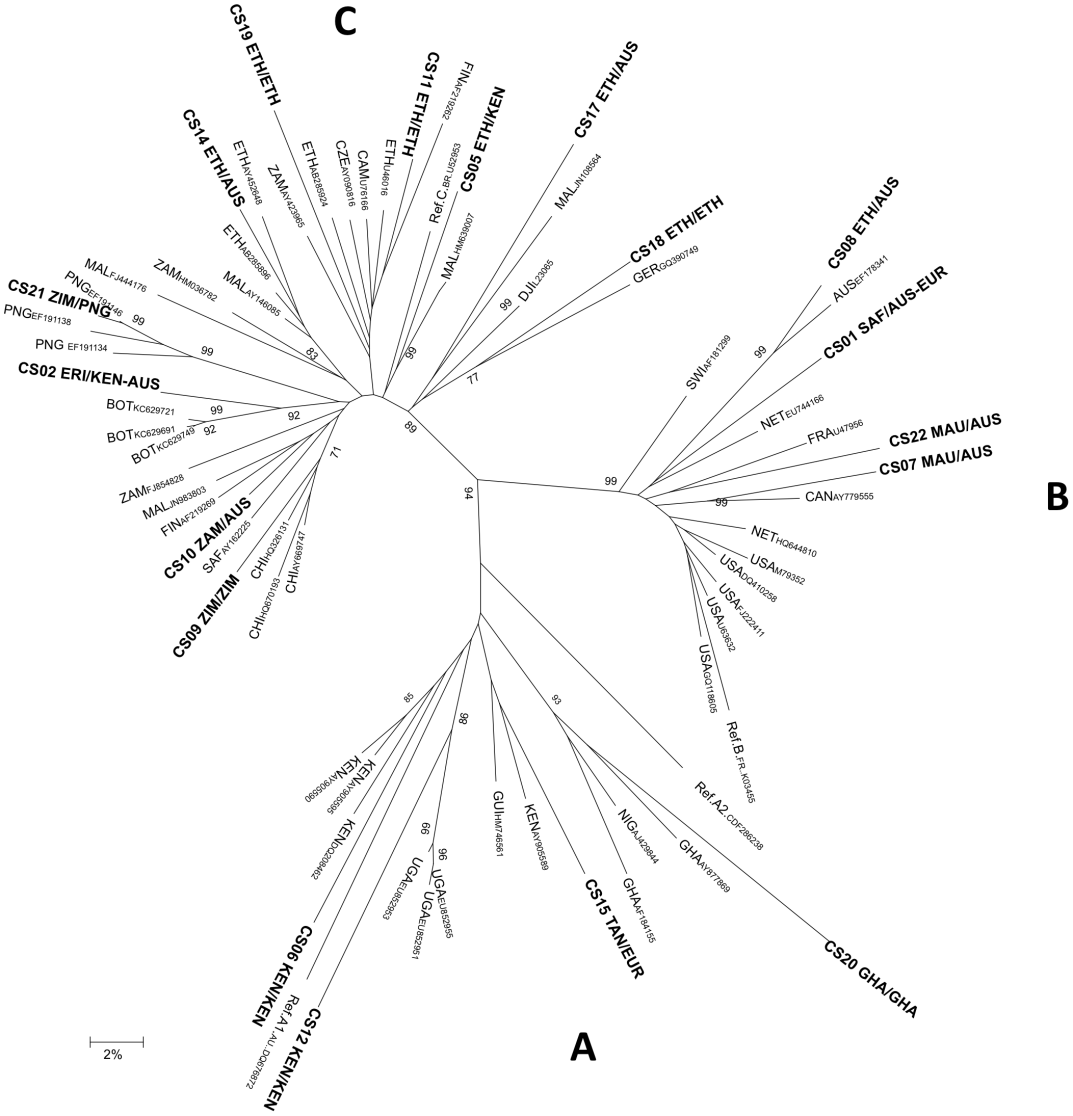
African-born Victorian residents with HIV: phylogenetic analysis of V3 env sequences and reference subtype A, B and C sequences. Neighbour joining tree reflecting the genetic relationship between HIV-1 isolates from African-born Victorians, the most homologous HIV-1 strains and selected reference strains. Study isolates are indicated in bold followed by Country of Birth/Country of reported exposure. Significant bootstrap values are indicated at the relevant node. Sequences were analysed over a 369bp region corresponding to the HIV-1 envelope region and spanning nucleotides 6984–7353 (HXB2 coordinates). The scale bar represents 2% genetic distance. *Country codes: AUS – Australia; BOT- Botswana; CAN – Canada; CHI-China; CZE – Czech Republic; DJI – Djibouti; ERI – Eritrea; ETH – Ethiopia; EUR – Europe; FIN- Finland; FRA – France; GER – Germany; GHA – Ghana; GUI – Guinea Bissau; KEN – Kenya; MAL – Malawi; NET – The Netherlands; PNG – Papua New Guinea; SAF – South Africa; SWI – Switzerland; TAN – Tanzania; USA – United States of America; 23 – Uganda; 24 – Zambia; 25 – Zimbabwe*.

The phylogenetic tree produced from the analysis of *env* sequence alignments is presented in Figure 1. Of the 18 cases with available *env* sequences, 15 clustered with reference sequences from the reported country of exposure. In the remaining three cases (CS10, CS14 and CD17), the *env* isolate clustered with reference sequences from the country of birth. Isolates from the three individuals identified with subtype A were all related to the “East African” cluster. The single CRF02_AG strain from Ghana (CS20) was linked to the “West African” A cluster. The four subtype B isolates grouped with other subtype B isolates from the USA, Australia and Europe; these isolates did not appear to cluster with any more specific geographic region. Nine of the ten cases with subtype C clustered with South-East African sequences, while the remaining sequence was closely associated with Papua New Guinean sequences, consistent with reports of heterosexual sex with a woman from PNG.

## Discussion

This small study shows how phylogenetic analysis may complement clinical data in ascertaining the location and circumstances of HIV exposure amongst African immigrants in Victoria, Australia. The results demonstrated a preponderance of non-B HIV-1 subtypes and a high degree of sequence diversity. Exposure histories were consistent with phylogenetic analysis. This study contributes to current understanding of the geographical and social complexities influencing the epidemiology of HIV in Victoria's African communities.

Most participants had non-B subtypes of HIV-1, particularly subtype C (the most commonly occurring subtype worldwide) or subtype A (distributed widely across Africa) [19]–[21]. However, little similarity was found between participant sequences within each subtype. These data contrast to findings from our earlier study of Vietnamese-born IDU in Australia, who were more uniformly infected with the Vietnamese subtype, CRF01_AE [25]. The predominance and diversity of non-B HIV-1 strains has been previously noted in African immigrant populations in Europe, Canada and Australia [22], [36], [51], [52], reflecting the huge genetic diversity of HIV within Africa [53].

The genetic heterogeneity of study sequences and diversity of reported exposures located participants within separate transmission networks. All but one of these networks appeared to be based in Africa, indicating the epidemiological continuity between African populations within Africa and the African diaspora [26], [54]. The exception was the *env* subtype C sequence of CS21, which appeared to belong to a transmission network based in PNG (the country of his reported exposure) rather than one based in his country of birth. This case illustrated the additional information obtained through phylogenetic analysis, beyond simple serological categorisation by subtype, although serological assays may be useful in preliminary screening of samples [10].

Heterosexual sex was the commonest exposure route for these African immigrant PLHIV, as it has been amongst African immigrants in the UK [10]. However four (20%) reported acquisition through male-to-male sex, highlighting the importance of this route of exposure in African populations within Africa and the diaspora [5], [9]. The association between subtype B and exposure through sex between men is consistent with historical patterns in Australia [51] and with a recent study of HIV infections diagnosed in Victoria 2005–2010; however, non-B subtypes of HIV-1 have been isolated from native-born and foreign-born MSM in studies from Australia and abroad, whilst “African” transmission networks unsurprisingly include individuals not categorised or self-identified as “African” [22], [55]. Subtype B sequences isolated from all four MSM participants resembled *env* subtype B sequences from Europe, Australia and the US, but were dissimilar to each other. This was consistent with the geographically diverse histories of sexual exposure and with existing evidence of multiple, superimposed, transnational sexual networks mainly (but not exclusively) including MSM [23], [26], [29], [35].

Just over half of the participants reportedly acquired HIV in Africa, but almost a third reported acquisition in Australia. The diverse exposure locations and high proportion (70%) diagnosed late, justify the inclusion of African migrants as a “priority population” in the Australian HIV response [1]. Timely diagnosis (for African settlers arriving in Australia with prevalent HIV infection) must be complemented by prevention, for other African Australians, of HIV exposure after migration. Health promotion about HIV in African communities should address the effect of migration-related HIV screening on subsequent risk perception in Australia [13]–[15].

This study is limited by its small size. However as the number of African-born people diagnosed with HIV in Victoria is not large, this sample size represents a substantial proportion of this population. Additional complexity arises from the high proportion of late diagnoses among African-born cases of HIV living in Victoria, which suggest that testing rates may be too low to provide a solid basis for estimates of HIV prevalence in Victoria's African communities [2]. This study provides no evidence for a locally based cluster of HIV transmission among African Australians living in Victoria, although demonstrating the absence of any such cluster would require a much larger study.

One strength of this study is the collection of geographical and temporal details exposure as well as detailed demographic information. Participation was likely influenced by the degree of engagement of eligible patients with their treating doctors. Ascertainment of details regarding HIV exposure was subject to recall bias and willingness to disclose sensitive information. The extent of participation also limited the samples available for sequencing. Sequencing confined to the V3 region of *env* limited the discriminatory power of the phylogenetic analysis and the ability to explore recombination [28]. However, genetic analysis in this study was used primarily to investigate the correlation between self-reported location of HIV exposure and genetic characteristics of HIV-1 strains circulating in the reported location of exposure. Limited sequencing of highly variable *env* region was probably sufficient for this purpose and has been successfully used in this context in previous molecular epidemiological studies [37]–[39]. The heterogeneity of sample *env* sequences and the absence of reported contacts between cases suggests that little additional epidemiological information would have been obtained by sequencing the whole genome.

Our study shows that international networks of social and sexual contact are likely to affect exposure to HIV for African Australians, within Australia and abroad. Phylogenetic data can complement existing surveillance methods in the development of more focused and effective strategies for HIV prevention and timely diagnosis of HIV infection in this mobile population.
